# The Foreign Journals: Surgery

**Published:** 1840-04

**Authors:** 


### SURGERY.

*On the Treatment of Malignant Ulcers by Cauterization with Corrosive Sublimate.*
By M. ORDINAIRE.

M. ORDINAIRE for the last fifteen years has been in the habit of employing the bichloride of mercury as a caustic in the treatment of cancerous, scrofulous, venereal, and other intractable ulcers, and has never observed the least symptom of absorption, which he ascribes to the instantaneousness of its destructive action on the absorbents, and to its being decomposed by the albumen of the tissues. The quantity of the powder employed varies with the thickness and the nature of the parts requiring to be destroyed, but never exceeds seven or eight grains. If the cancerous ulceration be superficial, a single application of caustic suffices; but it is generally necessary to repeat it after the separation of the eschar. He also employs this salt for the cauterization of strictures of the urethra. [The following, the third case related, is a specimen of the activity of this surgeon's practice:] M. I., innkeeper, subject from infancy to prolapsus of the gut, began at ætat twenty to experience much difficulty in reducing it after defecation, which increasing till his thirty-second year, reduction then became impossible. M. O. on examining the patient at this period, found at the orifice of the rectum a tumour as large as an egg, traversed by several deep sulci, dividing the mass into ulcerated lobes. The tumour was hardly tender to the touch, yet caused severe lancinating pain; it bled when handled, and discharged a very fœtid ichorous matter. Recognizing cancerous degeneration, the author removed with a bistoury the mass protruding beyond the anus, and having ascertained that diseased excrescences extended two inches up the rectum, determined on removing them with the bichloride. He prepared a cylindrical plug of shredded lint of the thickness of the finger and four or five inches long, smeared it with a glutinous matter, then sprinkled it all over with the caustic, and pushed it two inches and a half up the rectum. Severe pain soon came on, and there was great difficulty in persuading the patient to bear the plug for a few hours. Sloughs as large as an almond separated on the fourth day with the aid of injections and hip-baths, and further application of the caustic was only required to two spots at the margin of the anus, which had a suspicious appearance. The patient was entirely cured thirty-two days after the first cauterization; the sphincters performed their office well. The operation was performed twelve years since, and the patient enjoys perfect health.

*Gaz. Médicale de Paris.* No. xlvi. 1839.

---

### *On the Treatment of Synovial Tumours by Subcutaneous Incision.* By M. Barthelemy.

This mode of treatment was suggested by the very slight local or general disturbance which has been found to succeed the sections of muscles even of considerable extent, when the skin over them is not divided. The operation consists in raising a fold of the skin near the tumour, and passing a long thin lancet-shaped knife under it to the left side of the swelling, which is then by a sweep of the instrument split into two portions. The instrument is then withdrawn by the same narrow aperture at which it entered, and care is taken that no air should pass along the track of the wound. The fluid which the cyst contains diffuses itself into the surrounding tissue and soon disappears, leaving it is presumed no chance of the relapses which are common after every other mode of treatment.

The operation has been performed with success and with no subsequent ill effect by MM. Barthelemy, Marechal, and Malgaigne.

*Gazette Médicale. Dec.* 7, 1839.

---

### *On Division of the Muscles of the Back in Cases of Lateral Deviation of the Spine.* By M. Bouvier.

They who maintain that contraction of the muscles plays the chief part in the greater number of lateral deviations of the spine, and that these deformities should in consequence be treated by tenotomy, are required to prove the affirmation of the three following positions: 1. That in the majority of lateral deviations of the spine, as in deformities really produced by muscular contraction, the muscles corresponding to the concavity of the curvature are rendered extremely tense, when an attempt is made to straighten the spine. 2. That the permanent retraction of these muscles is not preceded in the greater number of cases by deformity of the spine. 3. That section or excision of these same muscles on the dead subject causes total or partial disappearance of the curvature. [M. Bouvier decides each of these points in the negative; and hence concludes that curvatures of the spine are not in the majority of cases capable of cure by the plan recently advised and practised by a rival orthopœdist of the French capital. The point at issue can, however, only be fairly settled by the results of the operation on the living subject.]

*L'Expérience,* No. cviii. *Juillet,* 1839.

*Cure of an Old Dislocation of the Humerus by Division of the Pectoralis Major, Latissimus Dorsi, Teres Major, and Teres Minor Muscles.* By Professor DIEFFENBACH.

HERR TH., a large landowner, upwards of thirty years old, had his right shoulder dislocated two years ago by a fall from his horse; the nature of the accident was not at first recognized, and afterwards, though all the usual means were adopted by several surgeons, the bone could not be returned to its place. The patient, therefore, came to Berlin; he was of a gaunt powerful form, with a pale complexion and but little fat, and his muscles were strong and prominent under the skin. The injured right shoulder was an inch higher than the left; the acromion formed a sharp angle; on the outer side the shoulder was deeply hollowed, and the scapula lay flat. The right arm was thinner than the left, and stood out far from the body. The head of the humerus lay on the anterior side of the chest, close to the clavicle, and two inches from the upper portion of the sternum. The patient had a constant sensation of cold in the limb, and the creeping which he had formerly felt had ceased. The pulse in the right radial artery was rather weaker than that in the left. The limb was useless, and only the hand could perform some slight actions.

By moving the arm in different directions, severe pain was produced in the part where the head lay surrounded by a thick wall of dense ligament into which it had worked itself. In drawing the arm outwards from the body, the pectoralis major, latissimus dorsi, teres major, and teres minor became tense with extreme pain. The last three of these muscles felt hard and tense, even when the arm was not drawn outwards. An attempt to reduce such a dislocation without dividing these muscles and the new joint would have been extremely dangerous, and had been found impossible; but (says the Professor) I anticipated success from the subcutaneous division of everything that resisted me.

The patient being placed on the table, with one folded sheet passed under the right axilla, and held by six assistants, another fastened round the right hand and held by six more, and a third round the upper part of the humerus held by three more (in the manner usually adopted by me in old luxations), the two first sets of assistants were ordered to pull against each other. I next bade them make a slowly increased extension, and then stop; I then passed a small scythe-shaped knife through the skin, and divided the most tense portion of the pectoralis major close to its tendon, which yielded with a cracking sound. I then again introduced the knife at the posterior border of the axilla, and divided one after the other the latissimus dorsi, the teres major, and the teres minor. All these muscles gave way with a cracking noise, which was increased by the resonance of the chest. I next passed my knife into three places by the head of the humerus, and divided in a similar manner under the skin the dense and hard false ligaments which surrounded the new joint, and lessening the extension, I loosened the head by a few rotations.

A powerful extension was now again commenced on both sides, and the three assistants behind the patient pulled suddenly while I conducted the humerus towards the joint into which it slipped on a sudden, without again springing out. One shoulder looked now just like the other. The thorax, the shoulder, and the arm were enveloped with bandages which were soaked with paste, and after a few hours they all became dry and hard, and prevented any motion of the right side.

The bleeding from the wounds, which were not larger than those made in phlebotomy, was at most a few drops. No unpleasant symptoms ensued, and the patient suffered even less than the majority of persons in whom I have reduced old dislocations. On the ninth day I took off the bandage; both shoulders had exactly the same level and form, and there was neither swelling nor pain. The punctures in the axilla had completely healed, and scarcely a trace of them could be found; there was no collection of blood or pus. The arm was already capable of motion, and its actions were far less hindered than they are sometimes after the reduction of recent dislocations; because in them there is often for a long time a sensitive contraction of the unnaturally stretched muscles, while in this case the division of the resisting muscles and of the newly-formed joint not only rendered the reduction possible, but at the same time diminished its after consequences. The limb is now again restored to perfect utility.

The Professor adds that he had lately occasion to reduce a luxation of the foot backwards of upwards of a year's standing, by dividing the tendo achillis, which forcibly drew the heel upwards. This limb also became useful again.

*Medicinische Zeitung. Dec.* 18, 1839.

---

*Case of Dislocation of the Elbow, in which the Radius and Ulna were thrown forwards, with Fracture of the Ulna.* By M. Richet.

A man fell from a high scaffold, as it was supposed, on his back and the palm of the left hand, and was admitted into the Hôpital St. Louis, in a state of partial collapse.

On examining the bend of the elbow, a hard oblong tumour was seen a finger's breadth above the condyles of the humerus, elevating the biceps and brachialis internus, and rendering the brachial artery comparatively superficial. This tumour was moveable on bending and extending the fore-arm, and rolled under the finger towards the radial side during supination and extension, giving rise to an indistinct crepitation. The olecranon was prominent, moveable transversely, but maintained its natural position. On tracing the edge of the ulna downwards, a flesh-wound was seen two fingers' breadth below the olecranon, through which the bone protruded. The condyles of the humerus, though preserving their natural relations with the posterior part of the ulna, projected considerably, so as to stretch the skin.

The dislocation being immediately recognized as that of the radius and ulna forwards, with fracture of the ulna, was easily reduced by extending the fore-arm, and then suddenly and forcibly bending it, at the same time forcing the upper extremities of the radius and ulna downwards and backwards. All deformity quickly vanished, the bones having regained their natural position, when, in consequence of extension having been remitted, an involuntary contraction reproduced the dislocation with the utmost ease. It was again reduced, and kept so, by means of a cushion, and bandage surrounding both fore-arm and arm—the fore-arm being bent at a right angle with the arm, and the cushion filling up the angle and keeping the bones in place—at the same time, a splint was applied along the inner side of the joint. The patient died three hours after, from the other injuries received.

On examination, it was found that the triceps was firmly attached to the whole of the posterior fragment of the ulna, as were also the extensor carpi ulnaris and anconæus, thus accounting for the natural position of the olecranon. A longitudinal fracture was seen, somewhat oblique, from above downwards, and from before backwards, running along the middle of the sigmoid cavity to the external border of the ulna, and thence backwards and inwards to a point a finger's breadth below the olecranon, which was the part that had protruded.

The ulna was thus divided into two fragments, the larger formed by the body, surmounted by the coronoid process; the smaller and posterior by the olecranon, and an inch and half of the ulna below it, ending in a point, which constituted the protrusion. The radius and ulna were seen on the front of the humerus, half an inch above its condyles. The capsular ligament was torn throughout, which explained the easy reduction and reproduction of the luxation.

[This case is worthy of remark, since it proves the possibility of a dislocation of the elbow forwards *without* fracture of the olecranon, contrary to the assertion of Sir A. Cooper and others. Boyer doubted the fact of its occurrence, even complicated with fracture of the olecranon, though he could conceive the possibility of it. Neither Petit nor Desault ever met with the case; and the only author who records such a form of dislocation as M. Richet has described, is Delpech, in his "Clinique Chirurgicale de Montpellier."]

*Archives Générales de Médecine. Dec.*, 1839.

*Memoir on the Ophthalmia reigning in Belgium.* By M. CAFFE.

THIS disease appeared in Belgium in 1814, but it is chiefly since 1830 that it has raged with such intensity as to have attacked an eighth of the soldiers, and in some regiments even the half. It has affected more than 100,000 individuals, and has deprived a considerable number of their vision, and left them chargeable to the state. A commission of Belgian practitioners, with the assistance of ophthalmic surgeons from Berlin and Vienna, failed in their endeavour to check the progress of this scourge, and in 1838 there were still 5000 ophthalmic patients in an army of about 50,000 men.

In its symptoms this ophthalmia does not appear to differ materially from those with which English readers are familiar in the works of Veitch and other writers on the Egyptian epidemic; the more important part of M. Caffe's memoir is contained in his investigations into its origin and mode of extension. Reviewing the various causes to which its raging in the army has been ascribed, the author shows that it cannot be attributed to the introduction between the eyelids of the pipe-clay used in cleaning the belts and other white leather accoutrements of the soldiers, or of the powder (*tripoli*) with which their buttons and copper ornaments are polished, because the ophthalmia has affected regiments which never use those substances, and has not shown itself among other European troops who employ them in large quantity. He proves also that it is not due to the diet of the soldier, the abuse of spirituous drinks, the too frequent cutting of the hair, fatigue, sudden suppression of perspiration, melancholy, chlorine-fumigations used in the treatment of itch or syphilis, nor to the unhealthiness of the lodgings, to which Jungken had too hastily assigned it. He refutes especially the idea of the *compressionists*, who, with M. Vleminckx, the inspector-general, hold that the ophthalmia is produced by the pressure of the veins of the neck by a hard stock and the collar of the coat, and of those of the forehead by an inelastic heavy cap. The inefficiency of these causes M. C. shows by the facts that the French uniform before 1830, though in these respects similar to the Belgian, never produced an epidemic ophthalmia, that even in Belgium certain regiments are unaffected though dressed like the others, that the recruits who have not yet suffered from compression are often attacked on their first joining a corps in which the disease is raging, and that a complete change in the soldiers' dress has not been followed by any decrease in the disease.

M. Caffe believes that the extension of the disease depends on its contagious nature, by which it is capable of communication either by direct inoculation, by the application of the matter secreted by the diseased eye on the fingers, the linen or fluids impregnated with it, to the previously healthy eyes, or by mediate contagion or infection of the air by miasmata arising from the evaporation of the secreted fluids. Its transmission by the immediate contact of the secretion of the inflamed eye is generally admitted. The inoculation of the ophthalmia has not only succeeded on dogs, cats, and guinea-pigs, but the same result has been obtained in man, by placing the virus on an opaque cornea, or when some one with too firm a faith in non-contagion has submitted healthy eyes to the same experiment. Instances of accidental inoculation are abundant in the records of the Belgian physicians, and some of the most remarkable are cited. M. Fallot states that on the 25th of January, 1834, there was not a single case of ophthalmia in the garrison of Namur; on that day two came into the hospital, and from that time the disease spread; two nurses were attacked and each lost an eye. A soldier went home with an ophthalmia from the hospital at Brussels. His father, previously well, was affected a few days after with a violent conjunctivitis, and his mother and five brothers and sisters all in succession contracted the same disease. Another soldier went home with ophthalmia from the same hospital, and immediately after his arrival his sister was attacked. In a third similar case, the soldier's mother, brother, and sister were affected, but his father and elder sister escaped. In a fourth, the father, mother, and five brothers and sisters were all attacked. These and many other equally well-marked examples seem to leave no doubt of the extension of the disease by direct contagion.

The miasmatic or indirect contagion is less evident; but if, says M. C., one considers the influence which an atmosphere charged with exhalations from the purulent matter must have on the conjunctiva, as well as the rapid propagation and speedy aggravation of the disease in ill-ventilated places, whatever precautions are taken against direct contagion, one will be disposed to attribute at least some share in the production of the epidemic to miasmatic infection.

The means which M. C. recommends to put a stop to the progress of the disease are such as naturally arise from the opinion which he has formed, and, as it would seem, justly, that this ophthalmia, though probably originating in and often aggravated by peculiar local circumstances, spreads from one individual to another by direct or indirect contagion. They are chiefly directed to the careful exclusion of all in whom the disease or a suspicion of it exists, from association with their comrades.

*Bulletin de l'Acad. Roy. de Médecine. Janv.* 15, 1840.

---

### *On the Cure of Congenital Squinting by Division of the Internal Straight Muscle of the Eye.* By Professor Dieffenbach, of Berlin.

[The following cases are masterpieces from the hand of a master. The operation is beautiful in its simplicity, and the result delightful to contemplate. Who shall set bounds to the progress of surgery? These operations are the first of the kind ever performed on the living subject; but Stromeyer has the merit of having suggested the operation, and he performed it on the dead body, with a direct view to proving its practicability on the living.]

Case i. The subject of this operation was a child seven years old, whose eye was drawn far into the inner angle of the eyelids, so as to produce considerable disfigurement. The operation was performed in the following manner: The head of the child was held against the chest of one assistant, while another with two hooks kept the eyelids widely apart. The operator then passed a third hook, which he gave to a third assistant to hold, through the conjunctiva, and to some depth in the subjacent cellular tissue at the internal canthus. He next fixed a fine double hook in the sclerotica at the inner angle, and taking it in his left hand drew the eye outwards. Then cutting into the conjunctiva close to the ball where it is continued from it to the internal canthus, and penetrating more deeply by separating the cellular tissue by the side of the sclerotica, he divided the internal rectus muscle close to its insertion with a fine pair of scissors. The eye was immediately drawn outwards by the external rectus, as if it had received an electric shock; and in another instant became straight, so that there was no difference perceptible between its direction and that of the other eye.

The hemorrhage during the operation was but slight though sufficient to impede it. The after-treatment consisted of cold lotions; no inflammation ensued, and within eight days the cure was completed.

Case ii. Carl Gerhard, æt. ten, affected with squint since his fourth year. His parents wishing him to become a printer, were anxious to have this defect removed as it interfered with composing. The right eye was so completely drawn into the inner angle that on a first view, the point of junction of the iris and sclerotica formed the centre of the anterior surface of the eyeball. By an effort the eye could be drawn from the canthus and placed straight, but could not be turned at all outwards. The operation was performed as in the last case, the conjunctiva being cut through, and the sclerotica laid bare to the extent of four lines, in order to bring the muscle into view, which was cut with a curved scissors as before. The squint was gone; the eyeball when at rest stood nearly straight, or rather a little turned outwards; and could be turned more readily by the patient's efforts in this direction than inwards. All the other movements of the eye were free. The bleeding was here much less than in the former case and caused no interruption. The sudden turning of the eyeball outwards, observed in the first case, did not take place here.

The boy felt quite well on the following day. He could separate the eyelids without difficulty. The conjunctiva in the inner angle of the eye was red. The eye was nearly straight, only turned a little more outwards than the other. In eight days the cure was complete and the eye quite straight.

CASE III. Albert Victor, æt. fifteen, affected with strabismus of the left eye since his earliest infancy. The eyeball was turned deeply into the inner angle, but by an effort of the will it could be turned straight, but on this effort being relaxed, it instantly returned to the former position. The operation was performed precisely in the same manner, it being only here specified that the external incision in the conjunctiva was semilunar, and that the muscle was cut by introducing the pointed blade of the scissors beneath it. As soon as the hook that held the eye was removed, the ball turned at first outwards, but in a moment returned to the straight position. The edges of the wound did not gape, so that the external incision was barely perceptible. The eye was covered with a cold poultice and the patient subjected to the antiphlogistic regimen. In eight days the cure was complete and the squint entirely gone.

*Medicinische Zeitung. Nov.* 13, 1839., and *Feb.* 5 and 12, 1840.

---

### *Cases in Autoplastic Surgery—Formation of New Eyelids.*
### By Dr. E. LABORIE.

THIS paper contains five successful cases of reparation of partially or completely removed eyelids, which have been observed by the author under the care of M. Jobert and M. Blandin.

In the first, of which the subject was a man æt. fifty-one, with a malignant tumour of the lower eyelid, M. Jobert removed it all from the external canthus to within two or three lines of the punctum lacrymale, exposing and removing some of the fibres of the orbicularis muscle. He then cut from the upper part of the cheek a flap of skin of an elongated triangular form, with its base slightly rounded and directed externally, and of which the truncated summit which was to serve as a pedicle was situated a little below the outer angle of the eye. This flap, which when it was dissected up was an inch and a half long, and half an inch wide at its outer part, was now twisted upon its pedicle and applied in the surface of the wound made by the removal of the part of the eyelid. It exactly fitted, and was fixed by three sutures. The wound on the cheek was also united by suture, and a simple slightly compressing dressing was put over the whole.

During the first few days after the operation, the wounds and the flap of skin swelled very much, but after this had ceased the adhesion was found complete. Notwithstanding two severe attacks of erysipelas, the reparation was in the end more perfect than could have been imagined; the punctum lacrymale having been preserved, there was no running of tears over the cheek, and the fibres of the orbicularis palpebrarum that remained gave the new eyelid a certain degree of natural motion.

The subject of the second case was a scrofulous woman of twenty-three, who was also under the care of M. Jobert, with ectropion of the left upper eyelid, which from cauterization and other severe treatment was in a completely incurable state. An incision an inch and a half long was made horizontally along the affected eyelid. A flap of skin of an oval form, and of the same size as the previous incision, was then cut out from the cheek. Its pedicle was at the level of the upper and outer part of the malar bone, and it was inclosed between two vertical lateral incisions and an inferior incision which was slightly rounded. This portion being dissected up was twisted on its pedicle, applied on its under surface between the edges of the incision in the eyelid, and retained there by three sutures and a simple dressing.

The operation succeeded completely; the eye, which was before constantly exposed, was fairly covered by the new eyelid, which by the actions of what remained of the orbicularis muscle performed all the natural motions. A second operation of a somewhat similar kind was subsequently performed with the same success for the reparation of a loss of substance in the situation of the eyebrow. In this case a portion of the skin from the temple, which was in part covered with hair, was taken for the flap; the hairs continued to grow, and by cutting them to a proper length they formed an excellent substitute for the lost eyebrow.

The third case was one of ectropion of the lower eyelid from a burn, which was treated in the same manner as the second with the same favorable result.

The fourth was that of a young woman who from an acute inflammation and sloughing had lost the whole of the left upper eyelid except its free border, which contained the eyelashes, the cartilage, and the meibomian glands, from which a sticky fluid constantly running had fixed this remnant of the upper eyelid to the edge of the lower. There was acute inflammation of the exposed surface of the eye.

M. Blandin cut a flap of skin from the forehead, of an oval form with its pedicle at the root of the nose. Then having cut off the edges of the ulcerated aperture, he turned down the flap over it, and fastened it to them by sutures. Notwithstanding a very severe attack of erysipelas, the flap adhered perfectly at all parts. The new eyelid had lashes, and even a slight power of motion, though the orbicularis had been entirely destroyed; for a portion of the frontalis muscle turned down with the flap of skin preserved its contractility, and by the transverse position in which its fibres were now placed, it enabled the eyelid to be slightly raised.

The last case was one of ectropion of the lower eyelid, which was treated like the second and third, by fitting a flap of skin into the space between the edges of an incision made along the everted part. It was successful, and the eye obtained almost complete protection from the new eyelid.

*Gazette Médicale. Janvier* 13, 1840.

